# Adsorption of Surface Active Ionic Liquids on Different Rock Types under High Salinity Conditions

**DOI:** 10.1038/s41598-019-51318-2

**Published:** 2019-10-14

**Authors:** Shilpa Kulbhushan Nandwani, Mousumi Chakraborty, Smita Gupta

**Affiliations:** Department of Chemical Engineering, Sardar Vallabhbhai National Institute of Technology, Surat, Gujarat India

**Keywords:** Core processes, Petrology

## Abstract

A new class of surface active ionic liquids (SAIL) have been reported to be a greener alternative to the conventional surfactants in enhanced oil recovery (EOR). These SAILs work efficiently under harsh salinity conditions encountered in the reservoir thereby recovering more additional oil during the tertiary oil recovery process. Adsorption mechanism of SAILs on different rock surface is however, not yet reported in the literature. This article highlights adsorption mechanism of three cationic SAILs having different headgroups, viz., imidazolium, pyridinium, pyrrolidinium, on different rock surfaces (crushed natural carbonate rock and crushed sandstone rock). All the SAILs studied here however had the same tail length and same anion (Br^−^) attached to it. XRD and XPS characterization techniques reveal that the crushed natural carbonate rock contains a substantial amount of silica, thus rendering it a slight negative charge. Static adsorption tests show that the retention efficiency on the natural carbonate type of rock for all the SAILs was lower than the conventional cationic surfactant, CTAB. The adsorption data obtained thereby was examined using four different adsorption isotherm models (Langmuir, Freundlich, Redlich-Peterson, and Sips). Results suggest that Sips adsorption isotherm model can satisfactorily estimate the adsorption of all the surface active agents on the natural carbonate rock. Factors like mineralogical composition of rock surface, presence of divalents, temperature, and structure of surfactants strongly affect the amount of surfactant adsorbed on reservoir rock. In order to evaluate the simultaneous effect all these factors as well as their interdependence on the retention capability of the three SAILs, a design of experiments approach has been employed further in this study. Statistical analysis of the data obtained after performing the full factorial experiments reveal that at high salinity, imidazoluim based SAIL show minimal adsorption on crushed natural carbonate rock at higher temperature. In general, at a given ionic strength, with increasing temperature as the amount of divalent in the aqueous solution increases, the amount of SAIL adsorbed on both the rock types decreases. Electrostatic attraction is the basic mechanism in governing adsorption of SAILs on the two types of rock surfaces. Results presented in this work can be used for EOR schemes.

## Introduction

In the oil industry, surfactants find application in different operations like drilling, hydraulic fracturing, demulsification, crude oil transportation, corrosion inhibition, waterflooding, chemical flooding, foam flooding and steam flooding^1–3^. Thus they are important both for the improvement of production economics as well as total recovery of petroleum. In a chemical enhanced oil recovery process (CEOR), surfactants can be used in surfactant flooding, alkali/surfactant/polymer flooding, surfactant/polymer flooding and foam flooding^4,5^. For all surfactant-based EOR process, one of the most important requirements is development of surfactant systems that have minimal chemical adsorption and low mechanical entrapment onto the reservoir rock^1,4,5^. Adsorption on the rock surfaces, as surfactant solution moves forwards, will decrease surfactant concentration in solution leading to an inefficient reduction of IFT, hence recovering lesser oil than expected. This may render them less efficient in practical applications of EOR^1,4,5^. An understanding of the adsorption behaviour of surfactant used in EOR processes is helpful for development of such applications. As a result many researchers have conducted static and dynamic adsorption tests for different types of anionic, cationic, nonionic, amphoteric and mixture of surfactants^6–16^. Adsorption of surfactants on solid surfaces is due to the complex interaction between the surfactant species or its complexes with the species on or near the solid surface^6^. Factors that affect adsorption of surfactant from a solution include solution pH, temperature, type of surfactant used and its concentration, the morphological and mineralogical characteristics of the rock, the type of electrolytes present in solution and their ionic strength and nature and concentration of co-surfactants and polymers present if added^4–6^. Most of the studies have been limited to adsorption of surfactants on rock surfaces under low salinity environment i.e. <5 wt.% total dissolved solids. However, actual reservoir salinities can range from 5–20 wt.% total dissolved solids comprising of about 0.04–1.0 wt.% divalent cations^17^. Since high adsorption of surfactant can impart inefficiency to the CEOR process and make it economically unfeasible, it is desirable to select surfactants having retention less than 1 mg/g of rock^18^. Most commonly used anionic surfactants, petroleum sulfonates and nonionic surfactants tend to adsorb easily on the reservoir rock, leading to low oil recovery in the tertiary process^4^. To overcome this situation, researchers have reported methods of minimising surfactant loss due to adsorption on reservoir rock by addition of sacrificial agents and selecting salt tolerant surfactants^19–22^.

Certain ionic liquids with long alkyl chains possess amphiphilic character owing to the distinct hydrophilic head and hydrophobic tail that determines their surface activity and the ability to self-organize as micelles. Such ionic liquids are known as surface-active ionic liquids (SAIL)^23–25^. Recently many researchers have evaluated the potential application of theses SAILs in EOR^26–39^. Results show that SAILs have many advantages over the conventional surfactants that are commonly used for this purpose, for instance: (a) SAILs do not lose their functionality under high saline and high-temperature reservoir conditions. (b)With the help of simple reactions structural changes in SAILs are possible, in principle to design task-specific SAILs for specific reservoir conditions. (c) Aqueous solutions of SAILs are more viscous as compared to the traditional surfactants, which is important to maintain favourable mobility ratio in surfactant flooding EOR processes. (d) Certain SAILs can alone reduce IFT between crude oil and aqueous solution to ultralow values without the need of co-surfactants. Use of such co-surfactants poses an environmental risk due to their high toxicity.

There are however, only a few experimental works studying the adsorption behaviour of SAILs on reservoir rock, available in the literature. Hezave *et al*. also performed three dynamic adsorption tests to evaluate adsorption of SAIL, C_12_mimCl on the rock surfaceduring core flooding experiments^27^. They investigated adsorption of C_12_mimCl on fresh core, core priory saturated with formation brine and on core priory saturated with both formation brine and oil. They revealed that the adsorption of IL solution on the rock surfaces is less when formation brine and oil are present in the system^27^. Bin Dahbag *et al*. performed dynamic adsorption test for SAIL, AMMOENG 102, to study the adsorption of the SAIL onto kaolinite (most commonly found in sandstone) rock^29^. It was observed that considerable adsorption takes place onto kaolinite surface at high salinity rather than low salinity^29^.

To get a more realistic data for the adsorption behaviour of SAILs, static adsorption tests are required to depict their adsorption isotherms at the solid-liquid interface. In this study, the adsorption behaviour of three SAILs, belonging to the pyridinium, pyrrolidinium and imidazolium based ionic liquids (having the same hydrophobic tail length and same anionic moeity attached); onto crushed natural carbonate rock and crushed sandstone rock has been evaluated. Different characterization techniques have been employed to determine mineralogical composition and surface charge of the adsorbent rock surfaces. The retention efficiency of the three SAILs onto crushed natural carbonate rock has been compared to that of an analogously structured, conventional cationic surfactant, Cetrimonium bromide (CTAB). Langmuir, Freundlich, Redlich-Peterson and Sips isotherms have been used to model the adsorption behaviour of each SAIL as well as CTAB. Design of experiments methodology has been employed to screen SAILs having minimal adsorption on the two adsorbents when subjected to varying conditions of temperature and composition of brine. Results presented in this work can be helpful in screening SAILs for surfactant-based EOR schemes.

## Theory: Adsorption Isotherms

An adsorption model relates the equilibrium surfactant adsorption at solid/liquid interface to equilibrium concentration of surfactant in the solution at a constant temperature. The four well-known general adsorption isotherms used in the present work to determine the equilibrium adsorption relation are described in this section, briefly.

### Langmuir adsorption isotherm

The Langmuir equation relates the amount of solid adsorbent adsorbed, Qe, to the equilibrium liquid concentration at a fixed temperature. The equation is expressed in its nonlinear form as follows:

1 $$Qe=\frac{{Q}_{{\max }}{K}_{L}Ce}{1+{K}_{L}Ce}$$

where *Qe* is the amount of adsorbate adsorbed (mg/g); *Q*_*max*_ is the maximum amount adsorbed (mg/gm); *K*_*L*_ is the Langmuir equilibrium constant (ml/gm) and Ce is the residual/equilibrium concentration after adsorption. This empirical model assumes that adsorption takes place at specific homogeneous sites and the adsorbed layer is one molecule in thickness. It assumes homogeneous adsorption such all sites possess equal affinity for the adsorbate, with no adsorption can take place at a site on which the solid adsorbate is already adsorbed^40,41^.

### Freundlich adsorption isotherm

Freundlich adsorption model assumes that the solute adsorption occurs on the adsorbent by multilayer adsorption. It assumes that adsorbent surface is composed of heterogeneous sites with non-uniform distribution of adsorption heat and energies. Amount of solute adsorbed per unit mass of adsorbent is a function of solute concentration. The nonlinear form of the Freundlich adsorption isotherm is expressed as:

2 $$Qe={K}_{F}C{e}^{1/n}$$

where, *K*_*F*_ (mg/g) and n are the Freundlich adsorption constants related to sorption capacity and sorption intensity, respectively^40,41^.

### Redlich-Peterson adsorption isotherm

Redlich–Peterson adsorption model incorporates features of both Langmuir and Freundlich isotherms. It is a three parameters model. This isotherm agrees with the Henry’s law and can be applied in either homogeneous or heterogeneous systems over a wide concentration range thereby amending the inaccuracies of two parameter, Langmuir and Freundlich isotherm equations^40,41^. The non-linearized form of Redlich-Peterson adsorption isotherm is given as follows:

3 $$Qe=\,\frac{{K}_{r}Ce}{1+\propto C{e}^{\beta }}$$

where Kr (ml/mg), α and β are Redlich-Peterson isotherm constants. At low concentrations, it approaches Langmuir adsorption isotherm (as the β value tends to one) and at high concentration it approaches Freundlich adsorption isotherm (as the β value tends to zero).

### Sips adsorption isotherm

Sips adsorption isotherm is a three parameter model and is also a combined form of Langmuir and Freundlich expressions. At low adsorbate concentrations, it reduces to Freundlich isotherm and bypasses the limitations associated with Freundlich isotherm model; while at high concentrations, it predicts a monolayer adsorption capacity characteristic of the Langmuir isotherm. The non-linearized form of Sips adsorotion isotherm is as follows:

4 $$Qe=\frac{{K}_{s}C{e}^{\beta s}}{1+{\propto }_{s}C{e}^{\beta s}}$$

where the three parameters Ks, α_s_ and β_s_ are Sips isotherm constants^40,41^.

In the present work, nonlinear regression technique is adopted in solving the above equations by maximizing the correlation coefficient between the experimental data points and theoretical model predictions with solver add-in function of the Microsoft excel^41^.

## Materials

### SAILs

In the present work cationic SAILs from three different families’ viz., pyridnium, pyrrolidinium and imidazolium were used. All the three SAILs had same hydrophobic tail length (n = 16) and the same anion attached to it (Br^−^). Chemical structure of the SAILs used in this study are given in Table 1. The adsorption behaviour of the three SAILs on crushed natural carbonate sample was compared with adsorption behaviour of conventional cationic surfactant, cetyltrimethylammonium bromide (CTAB) for the same adsorbent. CTAB also has the same chain length and same anion attached as in the three SAILs studied here. N-hexadecyl pyridinium bromide (C_16_PyBr) and CTAB were purchased from Sigma Aldrich. 1-hexadecyl-3-methyl imidazolium bromide (C_16_mimBr) was prepared and purified by the procedure mentioned elsewhere^42^. N-hexadecyl-N-methyl pyrrolidinium bromide (C_16_MPrBr) was synthesised according to the procedure reported by Goossens *et al*.^43^.

**Table 1:** Chemical structure of SAILs used in the study.

Ionic Liquid	Notation	Chemical Structure	Molecular weight (g/mol)	Melting point
N-hexadecyl pyridinium bromide	C_16_PyBr		384.44	64.5 °C
N-hexadecyl-N-methyl pyrrolidinium bromide	C_16_MPrBr		390.48	70 °C
1-hexadecyl-3-methyl imidazolium bromide	C_16_mimBr		387.45	68 °C

### Inorganic salts, solvent and dye

Sodium chloride (NaCl), Calcium Chloride (CaCl_2_) and chloroform (A.R grade) were purchased from Finar Chemicals. Aqueous solutions of the SAILs and brines were prepared by using degassed Millipore-grade water. Three types of brine solutions were prepared for adsorption studies carried out in this work. Each of the brines contain 15 wt.% of total dissolved solids (TDS). Composition of all the brines used in the present study is given in Table 2. ORANGE II dye (anionic dye) was purchased from TCI chemicals. Stock solutions containing 10^−4^ M ORANGE II dye were prepared in distilled water and stored in a dark bottle.

**Table 2:** Composition of brine solutions used in the present study.

Type of Brine (15 wt.% TDS)	Composition	Ionic strength (mol/l)
Brine I	100% NaCl	2.57
Brine II	95% NaCl and 5% CaCl_2_	2.64
Brine III	83% NaCl and 17% CaCl_2_	2.82

### Adsorbents

The crushed natural carbonate and sandstone used in this study were procured from RMR microminerals, Jaipur, Rajasthan. The samples were washed with double distilled water several times and then dried in an oven at 110 °C for 24 hrs.

### Experimental methodology

This section explains the procedure followed during characterisation of crushed carbonate as well as sandstone rock, static adsorption tests, quantitative analysis of SAIL in supernatant, determination of surface charge for each type of adsorbent and the procedure followed while implementing Design of Experiment methodology in order to analyse effect of four main factors having mixed levels on the response i.e. amount of adsorbent adsorbed (mg/m^2^). In general, each adsorption test was performed at least twice. Replicates showed minimum deviation (<1%) and average values of replicates for each particular condition are used in this study.

### Characterisation of adsorbent

XRD pattern of crushed carbonate and sandstone samples were obtained employing Rigaku D/Max 2200 × -ray diffractometer. X-ray Photoelectron Spectroscopy (XPS) was performed on the adsorbent materials using a scanning XPS micro-focused, monochromatic x-ray beam (PHI 5000 VersaProbe III, Physical Electronics, Inc). Specific surface area of the two adsorbent samples was determined using Thermo scientific Gas adsorption Porosimeter by applying N_2_ physisorption at −196 °C based on Brunauer-Emmet-Teller (BET) method.

### Determination of PZC of crushed carbonate and sandstone sample

PZC of both the samples was determined using powder addition/pH-drift method^44^. To a series of 25 ml glass bottles, 10 ml brine solutions (BRINE I, II & III) was added. The initial pH was adjusted from 2–10 with 0.5 M HCl and 0.5 M NaOH. A fixed amount of adsorbent (crushed carbonate or sandstone, 1 gm) was added in each of the bottles, capped and equilibrated for 24 hrs at 40 °C and 60 °C. The final pH minus the initial pH (ΔpH) was plotted vs. initial pH. The point where ΔpH = 0, is considered as the PZC for that particular adsorbent.

### Static adsorption test

Adsorption behaviour of SAILs and CTAB on carbonate samples were determined by static adsorption tests. These experiments were carried out for all the three SAILs and CTAB with different initial concentration, ranging from 0.1–6.5 mg/ml. SAIL/CTAB were dissolved in 20 ml of Brine I solution. After that 1.5 gm of carbonate sample was added to the SAIL solution in a conical flask. The mixtures of carbonate sample and surfactant solutions were then agitated by constant shaking at 40 °C for 24 hrs on a temperature controlled orbital shaker incubator (REMI, Model No. CIS-24 BL) at 110 rpm speed. After 24 hrs, the samples from the conical flask were transferred to centrifuge tubes. All mixtures were then centrifuged at a speed of 4000 rpm for 15 min using REMI centrifuge instrument (Model No. REMI R-23). After centrifugation the supernatant fluid was separated and analysed for the equilibrium concentration of SAIL/CTAB in the bulk solution after adsorption on carbonate sample.

### Quantitative analysis of SAIL/CTAB in supernatant

**Figure 1:**
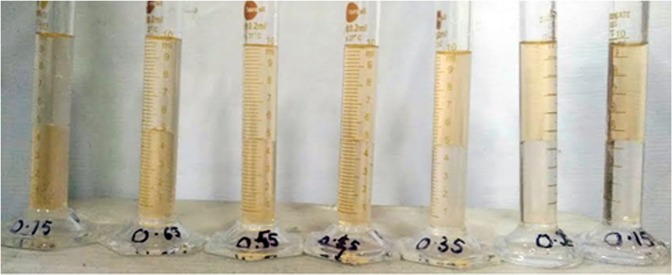
Colour change observed in chloroform (lower layer) on dissolution of soluble complex formed when the anionic dye reacts with the cationic SAIL/CTAB. Picture taken when making calibration curve for a SAIL (SAIL concentration in aqueous solution varying from 0–0.75 wt.%, here shown from 0.15–0.75 wt.%).

The equilibrium concentration of the SAIL/CTAB solutions were then estimated after proper dilution with the help of a two phase titration technique described by Few and Ottewill^45^. In this technique, 4 ml of diluted (1000 times) supernatant solution, 1 ml. of dye solution and 5 ml. of chloroform were pipetted into clean glass-stoppered test tubes. These tubes were then vigorously shaken. The anionic dye reacts with the cationic surfactant forming a complex which is insoluble in water (upper layer) but is soluble in the lower layer of chloroform and imparts an orange colour to it (Fig. 1). 4 ml of the lower phase was then transferred to the spectrophotometer cells to determine absorbance using UV-vis spectrophotometer. The dye in aqueous solution and the complex in chloroform solution both had absorption maxima at 485 nm. The amount of surface active agent adsorbed on the crushed carbonate sample, Qe (mg/g), was calculated by a mass balance relation:

5 $${\rm{Q}}{\rm{e}}\,({\rm{m}}{\rm{g}}/{\rm{g}}{\rm{m}})=\frac{(Co-Ce)\times V}{m}$$

where, Co and Ce are the initial and equilibrium concentrations of surface active agent (mg/ml) respectively, V is the volume of the SAIL solution (20 ml) and m is the weight of the carbonate sample (1.5 gm).

### Design of experiment

In order to evaluate the effect of the type of SAIL, Type of brine, Type of adsorbent and Temperature on the amount of SAIL adsorbed, a full factorial scheme of experiments have been designed. Full Factorial experiments help in evaluating the effect of each factor on the dependent/output/response variable (here, amount of SAIL adsorbed on adsorbent (mg/m^2^)). It also allows the researcher to evaluate the combined effect of two or more independent variables on the output variable, instead of the traditional “One factor at a time” methodology. This research methodology is termed as Design of Experiment (DOE) methodology. To achieve this, experimental work had been designed according to a Full Factorial Matrix containing four main factors with mixed levels attributing to about 36 runs. The factors studied and their respective levels are mentioned in Table 3. The outlays of all the tests carried out have been presented in Table 4. The amount of SAIL adsorbed on the adsorbent, was calculated by a mass balance relation

6 $${\rm{A}}{\rm{m}}{\rm{o}}{\rm{u}}{\rm{n}}{\rm{t}}\,{\rm{o}}{\rm{f}}\,{\rm{S}}{\rm{A}}{\rm{I}}{\rm{L}}\,{\rm{a}}{\rm{d}}{\rm{s}}{\rm{o}}{\rm{r}}{\rm{b}}{\rm{e}}{\rm{d}}\,({{\rm{m}}{\rm{g}}/m}^{2})=\frac{(Co-Ce)\times V}{m\times a}$$

where, Co is the initial concentration of SAIL (kept constant at 6.5 mg/ml), Ce is the equilibrium concentrations of SAIL (mg/ml) in the bulk solution after 24 hrs, V is the volume of the bulk solution (10 ml) and ‘m’ is the weight of the adsorbent sample (1 gm) and ‘a’ is the specific surface area of the adsorbent (m^2^/gm).

**Table 3:** Factors and their levels studied in design of experiments.

Factor	Type of SAIL (A)	Type of Brine (B)	Temperature (C)	Type of Adsorbent (D)
Level				
1	C_16_PyBr (Pyridinium)	BRINE I	40 °C	Sandstone
2	C_16_MPrBr (Pyrrolidinium)	BRINE II	60 °C	Carbonate
3	C_16_mimBr (Imidazolium)	BRINE III

**Table 4:** Outlay of the static adsorption tests carried out in accordance with the mixed level full factorial design.

TEST NO.	Type of SAIL (A)	Type of brine (B)	Temp. (C)	Type of adsorbent (D)	Amount of SAIL adsorbed (mg/m^2^)	Predicted values of amount of SAIL adsorbed from model equation (mg/m^2^)	Residual error
1	Pyrrolidinium	BRINE III	40	sandstone	10.002	10.33	−0.33102
2	Imidazolium	BRINE II	40	carbonate	1.399	1.778	−0.3792
3	Pyridinium	BRINE III	60	carbonate	3.042	3.498	−0.45598
4	Imidazolium	BRINE III	40	carbonate	0.921	0.9929	−0.072366
5	Pyrrolidinium	BRINE II	60	sandstone	10.598	10.51	0.08385
6	Imidazolium	BRINE III	40	sandstone	1.907	1.899	0.0078333
7	Pyrrolidinium	BRINE I	40	carbonate	7.282	6.985	0.2973
8	Pyridinium	BRINE II	60	carbonate	4.319	4.606	−0.2868
9	Imidazolium	BRINE I	40	carbonate	1.345	1.711	−0.3657
10	Pyridinium	BRINE II	40	sandstone	11.636	11.97	−0.33397
11	Pyrrolidinium	BRINE II	60	carbonate	5.428	5.389	0.03925
12	Pyridinium	BRINE I	40	carbonate	7.148	7.001	0.1471
13	Imidazolium	BRINE II	40	sandstone	3.955	3.445	0.5103
14	Pyrrolidinium	BRINE I	60	carbonate	5.905	6.219	−0.3148
15	Pyridinium	BRINE II	60	sandstone	10.17	9.875	0.2948
16	Pyrrolidinium	BRINE III	60	sandstone	9.491	9.263	0.2281
17	Pyridinium	BRINE III	40	sandstone	9.950	9.726	0.2239
18	Imidazolium	BRINE II	60	sandstone	1.225	1.782	−0.5566
19	Pyrrolidinium	BRINE III	40	carbonate	5.958	5.907	0.05083
20	Pyridinium	BRINE III	40	carbonate	5.277	5.156	0.1208
21	Pyrrolidinium	BRINE II	40	carbonate	6.646	6.771	−0.1248
22	Pyridinium	BRINE III	60	sandstone	8.116	8.005	0.1113
23	Imidazolium	BRINE III	60	carbonate	0.07077	−0.2338	0.3046
24	Pyridinium	BRINE I	60	sandstone	11.49	11.302	0.1878
25	Imidazolium	BRINE III	60	sandstone	0.3699	0.6099	−0.24002
26	Imidazolium	BRINE II	60	carbonate	0.6019	0.1764	0.4255
27	Imidazolium	BRINE I	40	sandstone	4.126	3.827	0.2991
28	Imidazolium	BRINE I	60	sandstone	2.760	2.781	−0.020604
29	Pyridinium	BRINE I	40	sandstone	12.296	12.78	−0.4838
30	Pyrrolidinium	BRINE II	40	sandstone	11.96	11.96	0.001652
31	Pyrrolidinium	BRINE I	40	sandstone	12.73	12.62	0.10597
32	Pyrrolidinium	BRINE I	60	sandstone	11.71	11.79	−0.08853
33	Pyridinium	BRINE I	60	carbonate	5.734	5.585	0.1489
34	Pyridinium	BRINE II	40	carbonate	6.965	6.639	0.3259
35	Pyrrolidinium	BRINE III	60	carbonate	4.951	4.899	0.05212
36	Imidazolium	BRINE I	60	carbonate	0.8143	0.7271	0.08721

## Results and Discussions

### Analysis of the crushed rock samples

**Figure 2:**
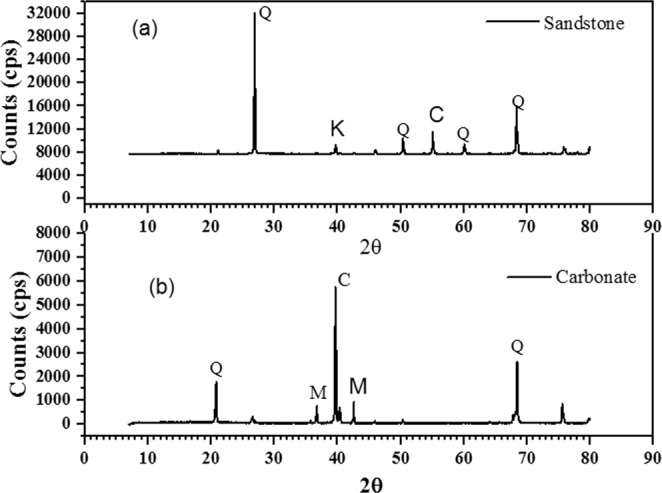
XRD of the crushed carbonate and sandstone sample (Q- quartz, K- kaolinite, M-Mg-Calcite and C-calcite).

**Figure 3:**
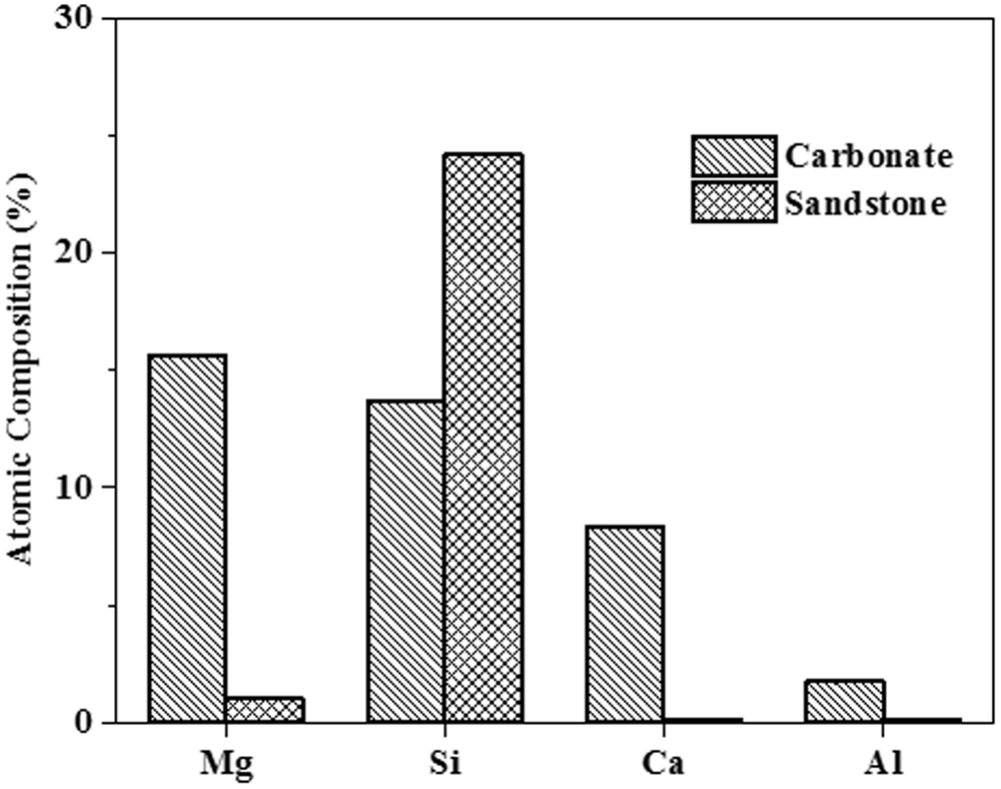
Atomic composition of crushed natural carbonate and sandstone rock measured by XPS technique.

The specific surface area of crushed carbonate rock and crushed sandstone rock calculated using the BET method was found to be 4.34 and 2.7 m^2^/gm respectively. X-ray diffraction (XRD) analytical technique was used to determine the mineral composition of the two adsorbents used in this work. The results obtained from this technique are shown in Fig. 2. The single headed peak indicates absence of any other phase. The X-ray diffraction analysis of the crushed sandstone is shown in Fig. 2(a). The characteristic peaks here are obtained at 2θ values of 21.1, 26.88, 39.72, 50.46, 55.2, 60.11 and 68.32. All these observed peaks correspond to the mineral quartz in common, though a very minor quantity of the kaolinite peak and calcite peak has also been noticed^33,46^. The X-ray diffraction analysis of the crushed carbonate sample is shown in Fig. 2(b). The characteristic peaks here are obtained at 2θ values of 20.8, 36.72, 39.6, 42.58 and 68.32. Interestingly unique quartz, calcite and Mg-rich calcite peaks have been obtained^33,46,47^. The atomic surface composition of the two adsorbent materials was determined using XPS characterization technique. Figure 3 shows the percentage atomic composition of calcium (Ca), magnesium (Mg), aluminum (Al) and silicon (Si) present in both types of adsorbent materials. It is evident from Fig. 3 that crushed sandstone contains primarily of silica and only small amounts of Ca, Mg and Al are present. However, it is seen that natural carbonate sample contains a substantial amount of Si, Mg and Ca. The XPS characterization technique further validates the result obtained from XRD analysis. Though carbonates are considered to be primarily composed of calcites, Mg-calcite and dolomites, presence of Si impurities might affect the conventional mineralogical properties of the crushed carbonate rock. Interestingly, Ekofisk field found Norwegian Sector of the North Sea is a fractured chalk reservoir but contains as much as 20% impurities, mainly silica^48^. Also Ma K. *et al*. in their studies on adsorption of surfactants on natural and synthetic carbonate materials pointed out presence of Si impurities in all the natural carbonate samples used by them^13^.

### Determination of PZC of crushed carbonate and sandstone sample

**Figure 4:**
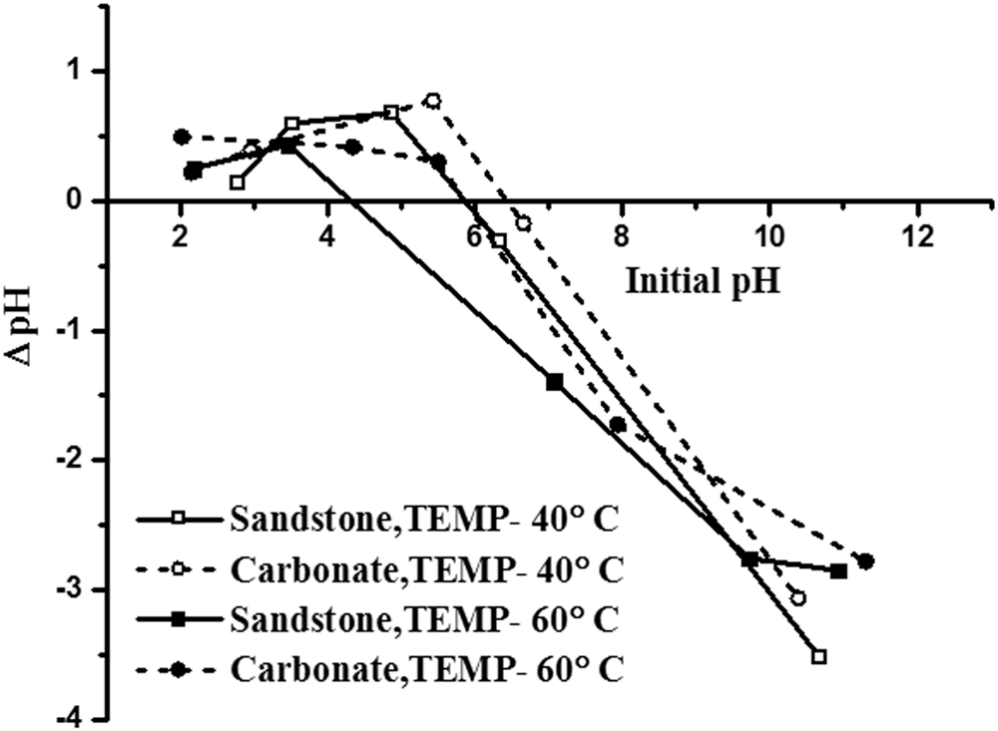
Temperature effect on the point-of-zero charge of carbonate and sandstone samples.

The rock surface charge is an important factor to be considered when studying adsorption of surfactants on it. When suspended in aqueous solution, a surface charge develops on the mineral particle due to preferential dissolution or due to hydrolysis of surface species^49,50^. The presence of monovalent and divalent ions in the brine, temperature and pH changes may create surface charges on the rock surface due to the ion exchange reactions. The pH at which the net charge of the rock surface is zero is called the point of zero charge^49,50^. PZC of a rock surface is an important interfacial property since the adsorption of different ionic surfactants on it is related to its PZC. In other words, with, if solution pH (unadjusted pH) in which the solid rock is suspended is below its PZC then the surface of the rock is positively charged and if the solution pH (unadjusted pH) is above its PZC then the surface of the rock is negatively charged. In the present study all the SAILs and CTAB are cationic in nature and it is important to determine the PZC of the adsorbents (crushed calcite and crushed sandstone sample) for establishing the reasons for adsorption. The PZC of adsorbents at different temperatures, when suspended in Brine I was determined by pH-drift method and is shown in Fig. 4. It was observed that as temperature increases from 40 °C to 60 °C, PZC for both type of adsorbent decreases. Similar findings were reported by M. Kosmulski in his study wherein it was observed that PZC value of alumina decreases with temperature^50^. The author further claimed that such a trend was common for oxides.

**Figure 5:**
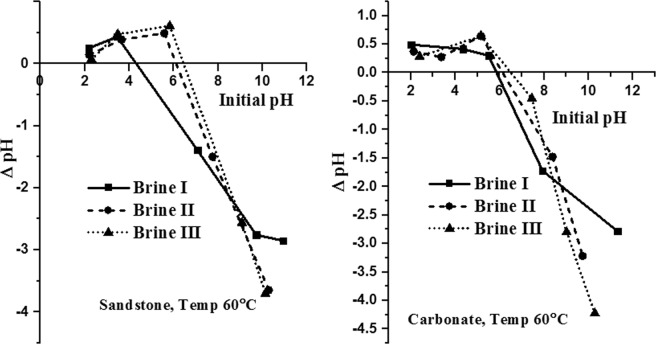
Point of zero charge plots for carbonate and sandstone samples in varying composition of brines.

The PZC of adsorbents when suspended in different types of brines was determined by pH-drift method and is shown in Fig. 5. PZC values of natural carbonate rock and sandstone rock has been displayed in Table 5. It can be seen that as the amount of divalent in the brines increase the PZC value for both the adsorbent increase. This might be due to presence of Ca^++^ ions which neutralize the negative charge on the surface and induce coagulation^51^. As seen from Table 5, for all brine compositions, both carbonate and sandstone rocks are negatively charged. As quartz (main component of sandstone) bears a negative charge for a long range of pH, sandstone samples studied here are more negatively charged as compared to the natural carbonate rock. Similar results were reported by many researchers wherein they found natural carbonates and limestone rock surfaces bearing a slight negative charge^13,51^. In general, carbonates are positively charged but presence of Si impurities influences its surface chemistry thus making it difficult to determine whether anionic or cationic surfactants should be used to minimize adsorption of surfactants on carbonate surfaces.

**Table 5:** PZC values of the two adsorbents in different type of brines at a constant temperature of 60 °C.

Type of adsorbent	Natural carbonate rock		Sandstone rock	
Type of brine	PZC (pH)	Natural (unadjusted pH)	PZC(pH)	Natural (unadjusted pH)
Brine I	5.86	6.271	4.3	6.853
Brine II	6.13	6.398	6.1	6.6
Brine III	6.51	6.64	6.43	6.557

### Static adsorption of three SAILs and CTAB on carbonate sample

**Figure 6:**
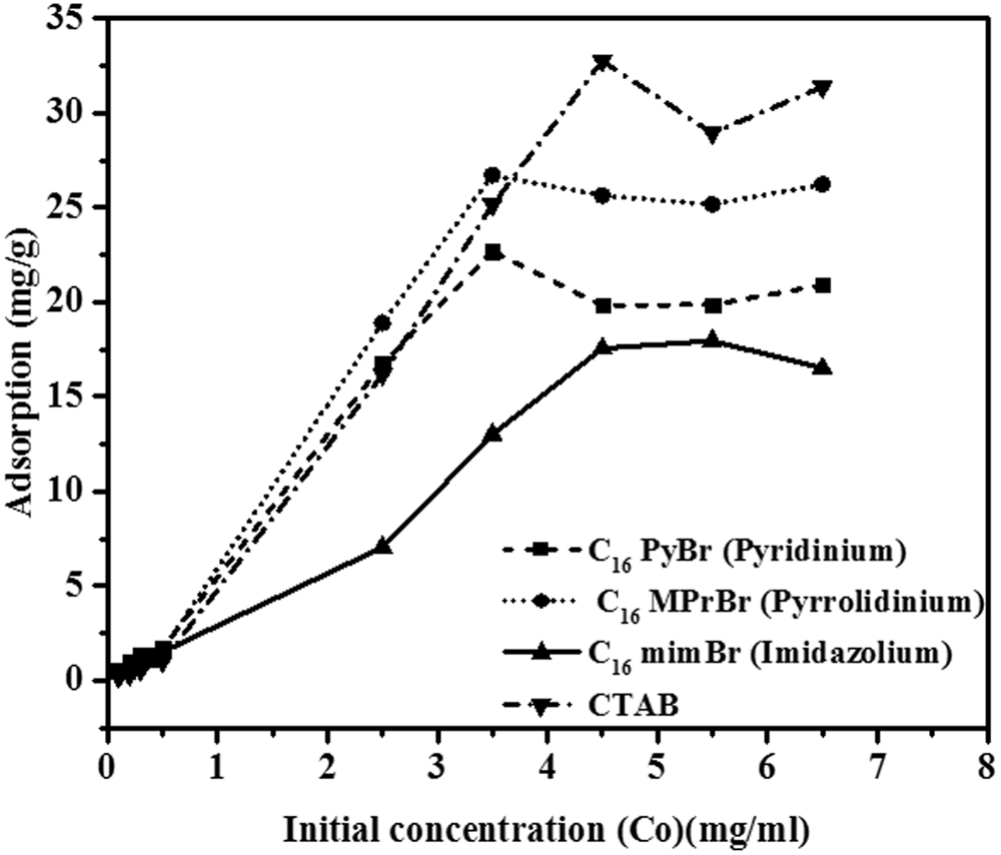
The adsorption of different types of surfactant onto crushed Berea sandstone as a function of initial concentration. (Temperature 40 °C and salinity 15 wt.%, BRINE I).

Figure 6 shows the adsorption of different types of surface active agents on carbonate samples suspended in BRINE I at 40 °C. It can be seen that adsorption of all SAILs and CTAB increases with initial concentration. Also significant retention for the SAILs as well as CTAB on carbonate rocks has been observed. Presence of Si impurities in carbonate rocks makes carbonate surface negatively charged which leads to adsorption of the cationic surface active agents (SAILs as well as CTAB) studied here. All the adsorption isotherms show a typical S-form. Similar behaviour was reported by many other researchers. Austad *et al*. in their study found that adsorption of ethoxylated surfactants on kaolinite displayed an S-shaped isotherm. In their studies they concluded that a typical S-form is developed on adsorption of negatively charged surfactants on oppositely charged adsorbents^8^. Sexsmith *et al*. also obtained an S-shaped isotherm for adsorption of cationic surfactant, CTAB on cellulosic fibre (negatively charged)^52^. From Fig. 6, it can be seen that the imidazolium based SAIL, (C_16_mimBr) was least adsorbed on the carbonate surface whereas the amount of conventional cationic surfactant, CTAB, adsorbed at the carbonate surface was greater than all the SAILs studied here. This behaviour might be attributed to the presence of a heterocyclic ring in the headgroup of all the SAILs studied here. CTAB, is a quaternary ammonium cationic surfactant that does not contain a heterocyclic headgroup. Properties of heterocyclic amines are strongly influenced by the presence of strain in the ring^53^ and hence it might be therefore that such SAILs adsorb less on the negatively charged carbonate surface. The maximum amount of a surface active agent adsorbed on the carbonate surface was in the order of CTAB > C_16_MPrBr > C_16_PyBr > C_16_mimBr.

**Figure 7:**
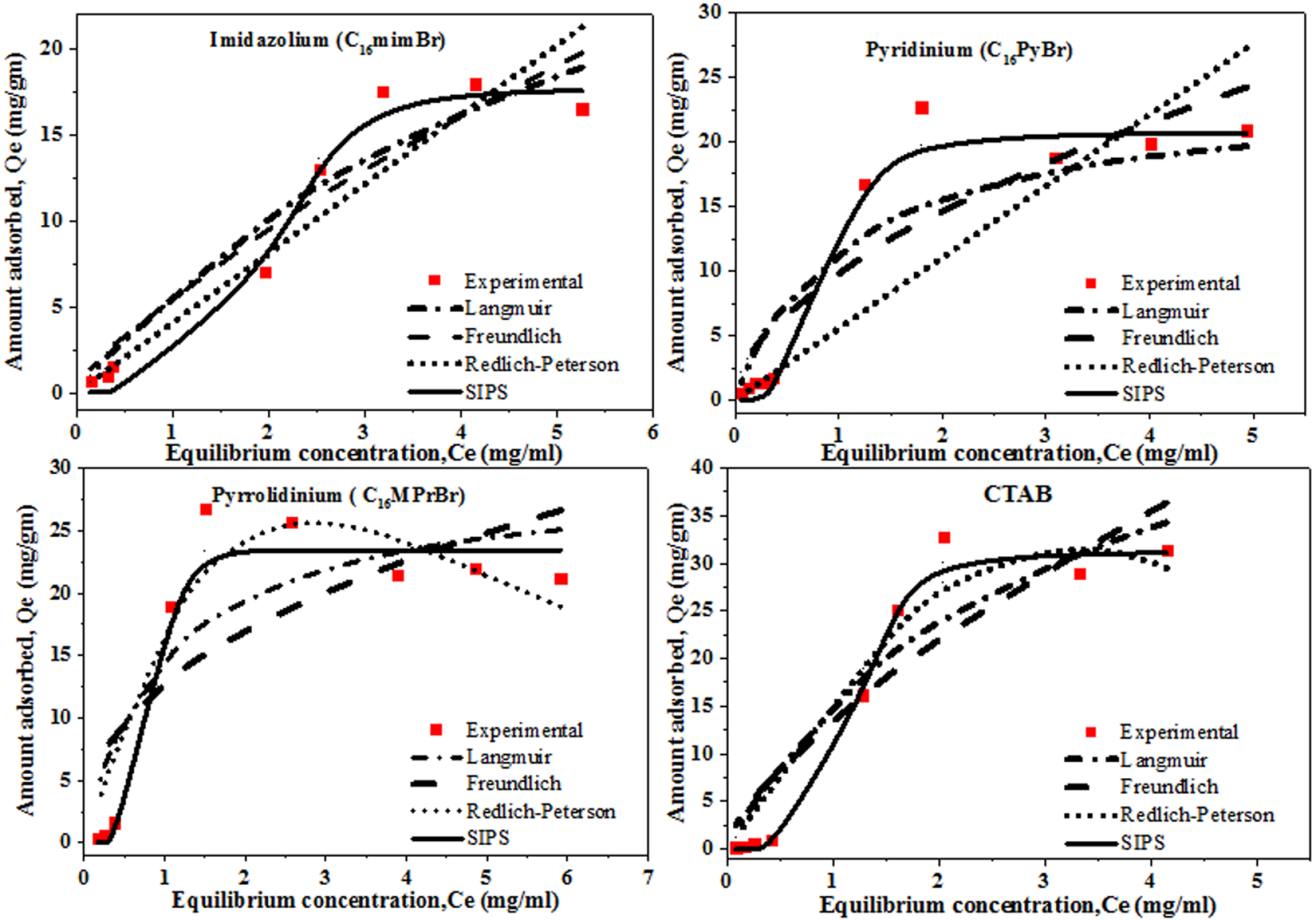
Curves representing fitting of Langmuir, Freundlich, Redlich-Peterson and Sips adsorption isotherms for adsorption of different SAILs and CTAB on carbonate sample. (Temperature 40 °C and salinity 15 wt.%, BRINE I).

In order to get a deeper insight of the adsorption behaviour of the three SAILs and CTAB studied here Langmuir, Freundlich, Redlich-Peterson, and Sips adsorption isotherm models have been used. Curve fittings for Langmuir, Freundlich, Redlich-Peterson, and Sips adsorption isotherm models for imidazolium (C_16_mimBr), pyridinium (C_16_PyBr) and pyrrolidinium and CTAB have been shown in Fig. 7. The regression coefficient (R^2^) as well as the parameters associated with each of the model have been calculated using solver add-in function of the Microsoft Excel and have been summarized in Table 6.

**Table 6:** Adsorption isotherm parameters of the three SAILs and CTAB.

Adsorption Isotherm	Langmuir	Freundlich	Redlich-Peterson	Sips
Surfactant				
C_16_MPrBr	Qmax = 29.09	K_F_ = 12.88	K_R_ = 18.67	Ks = 5.59
	K_L_ = 1.05	n = 2.45	α = 0.11	α_s_ = 0.24
	R^2^ = 0.81	R^2^ = 0.64	β = 2.15	β_s_ = 36.04
			R^2^ = 0.95	R^2^ = 0.97
C_16_mimBr	Qmax = 39.57	K_F_ = 5.7	K_R_ = 4.04	K_s_ = 0.11
	K_L_ = 0.17	n = 1.34	α = 0	α_s = _0.006
	R^2^ = 0.93	R^2^ = 0.91	β = 0	β_s_ = 6.8
			R^2^ = 0.87	R^2^ = 0.99
C_16_PyBr	Qmax = 23.61	K_F_ = 10.07	K_R_ = 5.52	Ks = 46.31
	K_L_ = 1.003	n = 1.82	α = 0	α_s_ = 2.24
	R^2^ = 0.92	R^2^ = 0.82	β = 0	β_s = _3.98
			R^2^ = 0.71	R^2^ = 0.99
CTAB	Qmax = 57.07	K_F_ = 13.65	K_R_ = 15.12	K_s_ = 5
	K_L_ = 0.36	n = 1.45	α = 0.01	α_s_ = 0.16
	R^2^ = 0.92	R^2^ = 0.86	β = 3.35	β_s_ = 7.4
			R^2^ = 0.96	R^2^ = 0.99

It can be seen that regression coefficient R^2^ value is maximum for Sips adsorption isotherm. Hence it can be concluded that the adsorption behaviour of all the cationic surface active agents, (SAILs as well as CTAB) follows the Sips adsorption model. According to D.G. Kinniburgh, Sips adsorption isotherm approaches an adsorption maximum at high concentrations and hence fits well into the S-form of the experimental adsorption profile of the three SAILs as well as CTAB^40^.

### Design of experiments

Loss of surfactants due to adsorption on reservoir rock imposes detrimental effects for otherwise efficient surfactant flooding processes. In future, more viable surfactant assisted EOR field projects will only be possible if the retention of surfactants upon reservoir rock is decreased drastically. Sandstone reservoirs contain a substantial amount of quartz (negatively charged). Hence to minimise loss of surfactant due to adsorption on negatively charged rock surfaces, anionic surfactants are preferentially used during surfactant flooding. Electrostatic repulsion between anionic surfactant and negatively charged quartz inhibits retention of surfactant on the reservoir rock. Because of the high adsorption of cationic surfactants on negatively charged sandstone minerals, cationic surfactants have not been used for field application in EOR processes for sandstone reservoirs but can be used in carbonate reservoirs (as carbonates are mostly positively charged). Presence of silica impurities might make the carbonate surfaces slightly negatively charged as observed in the present study. Formation brine present in all oil reservoirs contains multivalent ions like Na^2+^, Ca^2+^ and Mg^2+^. These multivalent ions adsorb on the negatively charged rock surfaces and may also reverse the sign of the surface charge^8,52^. In such cases when a surfactant slug containing anionic surfactant is injected into the reservoir, loss of surfactant occurs due to precipitation leading to low oil recovery. However, in the presence of the same multivalent ions, cationic surfactants do not precipitate due to electrostatic repulsion and minimal loss of surfactant takes place^19^. In view of the above discussion, in order to determine the amount of SAIL adsorbed on the negatively charged natural carbonates and sandstone, in presence of increasing multivalent ions and temperature has been studied in the present work. Design of experiment (DOE) approach has been employed to study the simultaneous effect of four independent variables (Table 3) on the amount of SAIL adsorbed (dependent variable). Experiments were designed in a way so as to facilitate examining all the factors at once instead of examining them individually as in the conventional experimental designs. A full factorial design matrix was created using MINITAB 17 statistical software (Table 4). The experimental runs in the matrix were executed and amount of SAIL adsorbed during each run was registered as response variable. To account for precision in statistical analysis, the experimental data as well as the predicted values and the subsequent residual errors, significant F-values, p-values have been reported upto 4–5 significant digits. MINITAB 17 software has been used to statistically evaluate the resulting experimental data, calculate adsorption means, determine interdependence of factors on each other and develop a mathematical model equation for predicting the amount of SAIL adsorbed.

**Figure 8:**
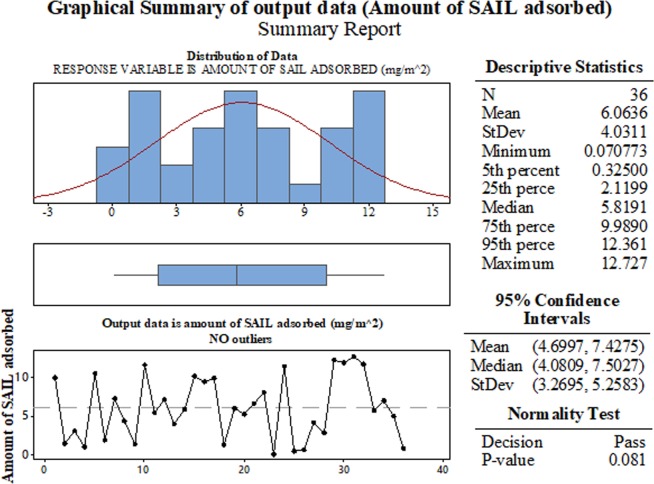
Graphical representation of the statistical information obtained for the output data (amount of SAIL adsorbed).

**Figure 9:**
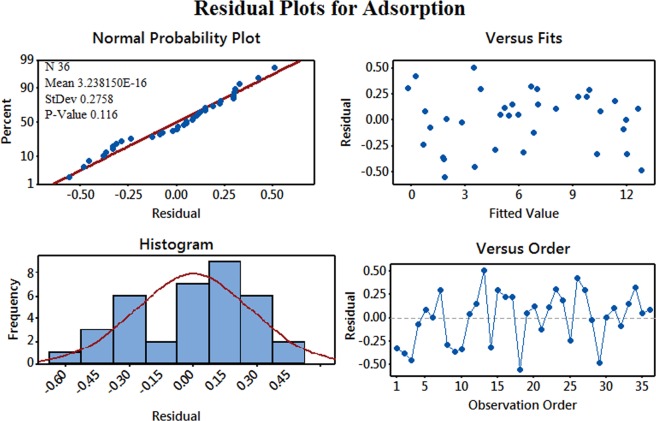
Residual plot for the output data (amount of SAIL adsorbed).

After data preparation and performing experimental studies, the next step is to obtain inferential statistics. In the present study, Minitab’s Graphical Summary has been used to obtain graphical as well as descriptive summary of the output data/response variable. In the present study the output data is actually amount of SAIL adsorbed during each experimental run. Figure 8 gives descriptive as well as statistical information about the output data. It shows spread of data and provides quick, visual summary of essential data characteristics. It shows a histogram of amount of SAIL adsorbed during the experimental runs, with an overlaid normal curve. As seen, the p-value (0.089) is greater than 0.05, which means that the output data is normally distributed and uniformly spread. Normal distributions are mathematically tractable. Also, there are no outliers (no red marks), which means none of the points in the output data is outside the range of expected values. Figure 9 shows a residual plot for the output data (adsorption). A residual plot helps to determine non-linearity, unequal error variances, and presence of outliers if any. The p-value is large (0.116). In addition, as seen in Fig. 9, the normal probability plot of the residuals fits the straight line indicating normal distribution. The histogram of the residuals represent general characteristics of the residuals including typical values, spread, and shape. Absence of long heavy tails in histogram of the residuals implies normal distribution. The graph on top right plots the residual terms against the fitted values. The residuals versus order plot helps to verify the assumption that the residuals are independent from one another (not correlated). The randomness of residuals is another indication that the model equation fits in well with the data.

**Figure 10:**
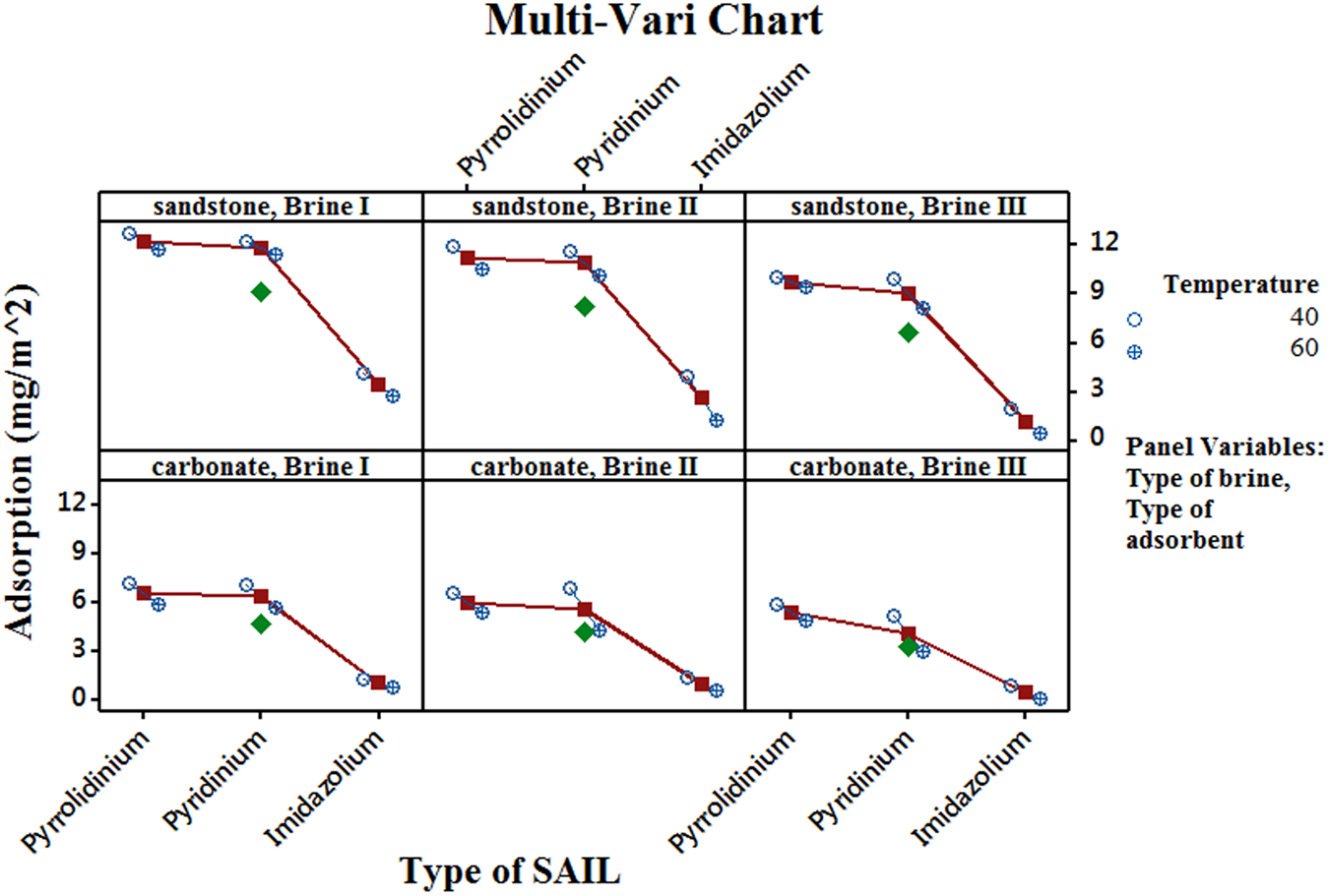
Multi-vari chart displaying trends of factors (Type of SAIL (**A**), Type of brine (**B**), Temperature (**C**) and Type of adsorbent (**D**)) affecting amount of adsorbate adsorbed.

Figure 10 shows the Multi-vari chart representing the data obtained for each run in a graphical form. Multi-vari charts provides a “visual” alternative to analysis of variance. These charts may also be used at the beginning of data analysis to know the trends for each factor at their respective levels. As observed in Fig. 10, imidazolium based SAIL display minimal adsorption tendency on both type of rocks, followed by pyridinium and pyrrolidinium based SAILs. Adsorption of all the SAILs upon both types of rock surfaces decreases as temperature increases and also as amount of divalents in the brine increases.

**Figure 11:**
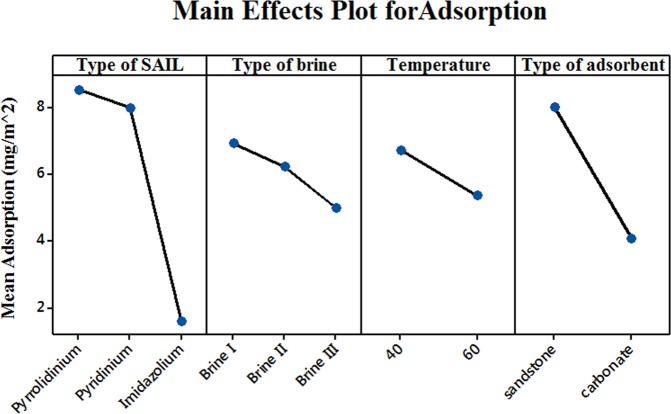
Main effects plot for mean adsorption displaying effect of each main factor on mean adsorption.

The main effect plot for the four factors viz., type of SAIL (A), type of brine (B), temperature (C) and type of adsorbent (D) is represented in Fig. 11. A main effects plot displays the adsorption mean for each factor level. Following conclusions can be made from the main effects plot:

- Imidazolium based SAIL (C_16_mimBr) display minimal adsorption on any type of rock surface as compared to pyridinium and pyrrolidinium based SAILs. Basically pyridinium and imidazolium possess an aromatic character (sp^2^ hybridised) while pyrrolidinium is sp^3^ hybridised and is non-aromatic. Also pyrrolidinum based ionic liquids have a higher disssociation constant as compared to imidazolium and pyridinium based SAILs. Hence water soluble pyrrolidinium SAILs get electrostsically atrracted towards the negatively charged adsorbents. The aromatic ring in pyridinium based ionic liquid is π-deficient wheras the aromatic ring in imidazolium based ionic liquid is both π-excessive and π-deficient (presence of two nitrogen atoms)^54^. When the surfactant contains π-deficient aromatic nuclei and the solid adsorbent has strongly negative sites, attraction between electron deficient aromatic nuclei of the adsorbate and negative sites on the adsorbent results in adsorption^55^. This results in more adsorption of pyridinium based SAILs as compared to imidazoliun based SAIL used in the present study.
- It can be seen that the mean adsorption decreases with temperature. Similar behaviour was reported by many researchers^14,56^. It has been reported that at high ionic strength, as temperature increases amount of surfactant physically adsorbed on the solid surface decreases.
  All the brines used in the present steady have approximately the same ionic strength.
- The main effects plot shows that the mean adsorption decreases as the amount of divalents in the brine increases. This result is in accordance with the statement mentioned above, presence of multivalent cations reduces adsorption of cationic SAILs on negatively charged rock surfaces. Also multivalent cations like, (Ca^++^) show a stronger tendency to compete for negative adsorption sites as compared to monovalent ions like (Na^+^) and sometimes may also reverse the sign of the surface charge^51^.
- Type of adsorbent used also has a significant effect on the mean adsorption. As seen from Fig. 11, minimal adsorption is obtained for crushed carbonate samples. From the PZC values obtained for crushed sandstone and crushed carbonate, it is clear that both of them bear a negative surface charge. Hence, positively charged SAILs would adsorb on both the adsorbents. As discussed earlier, sandstone samples are more negatively charged as compared to carbonate samples. Hence less adsorption of SAILs is observed for crushed carbonate samples.

**Figure 12:**
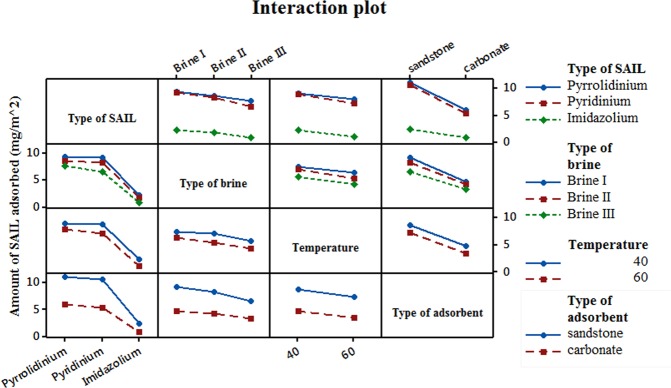
Effect of interaction among the four main factors (Type of SAIL, Type of Brine, Temperature and Type of adsorbent) on mean adsorption (mg/m^2^). Parallel lines for any two main factors indicate no interaction. Non – parallel lines indicate that the interaction between the two factors effects the output (here adsorption).

Interdependence among factors and their combined effect on amount of adsorbate adsorbed can be easily studied from the interaction plot. An interaction plot is a plot of means for each level of a factor with the level of a second factor held constant and enables us to study the two-way interaction between all the factors associated in the full factorial design. A matrix of interaction plot for adsorption has been presented in Fig. 12. Parallel lines in an interaction plot indicate no interaction. Non-parallel lines indicate that there is interaction between type of SAIL and all the other factors studied here and they have a combined effect on the amount of adsorbate adsorbed. Existence of parallel lines for all the other factors when considering two-way interaction indicates no interdependence on each other. However, the interaction plot doesn’t tell if the interaction is statistically significant and can be determined from the ANOVA table shows that statistically.

Significant main effects and interaction between factors were determined from analysis of variance (ANOVA) for adsorption means. An ANOVA table includes F-statistics and p-values, which are used to determine whether main factors or their interactions significantly effect the dependent variable. If the p-value is lower than 0.05, then that factor or particular, 2-way interaction is significant. ANOVA table generated after statistically evaluating the response in this study has been presented in Table 7. It can be seen that all factors studied here have p < 0.05 and hence are statistically significant in evaluating the amount of SAIL adsorbed. From the interaction plot in Fig. 12 it can be seen that the two-way interaction among some factors result in non-parallel lines. This implies that the interaction among these factors effect the amount of SAIL adsorbed. However, not all two way interactions have p-value < 0.05. The significant two-way interactions are: Type of SAIL × Type of brine and Type of brine × Type of adsorbent. In other words, interaction among these factors also effect the amount of SAIL adsorbed. A general regression for the complete model was performed using MINITAB 17 software. Regression in MINITAB 17 uses the ordinary least squares method to derive the model equation/prediction equation by minimizing the sum of the squared residuals. The regression equation is given in Supplementary Information S1. R-sq, R-Sq (pred) values obtained for the model equation are 99.53% and 97.63% respectively. Large value of predicted R-sq proves that model equation fits in well with the experimental data and have better predictive ability.

**Table 7:** ANOVA table for response, amount of SAIL adsorbed.

Source	DF	Adj SS	Adj MS	F-Value	P-Value
Model	19	566.065	29.793	178.79	0
Linear	6	535.338	89.223	535.45	0
Type of SAIL	2	356.477	178.239	1069.65	0
Type of brine	2	23.147	11.573	69.45	0
Temperature	1	16.962	16.962	101.79	0
Type of adsorbent	1	138.751	138.751	832.68	0
2-Way Interactions	13	30.727	2.364	14.18	0
Type of SAIL x Type of brine	4	1.398	0.349	2.1	0.129
Type of SAIL x Temperature	2	0.658	0.329	1.98	0.171
Type of SAIL x Type of adsorbent	2	25.837	12.919	77.53	0
Type of brine x Temperature	2	0.581	0.29	1.74	0.207
Type of brine x Type of adsorbent	2	2.245	1.122	6.74	0.008
Temperature x Type of adsorbent	1	0.009	0.009	0.05	0.822
Error	16	2.666	0.167		
Total	35	568.731			

## Conclusion

This work provides fundamental information on the adsorption behaviours of pyridinium, pyrrolidinium and imidazolium based cationic SAILs having the same alkyl chain length and anion attached to it. All the three SAILS studied here are potential candidates for surfactant flooding in highly saline carbonate reservoirs. Hence evaluating their adsorption behaviour on carbonate rock is a crucial study that needs to be done before using these SAILs in field. Comparative analysis with the adsorption behaviour of a conventional cationic surfactant CTAB has also been done. The experimental results and consequent analysis revealed some important features of adsorption behaviours:

1. The conventional cationic surfactant showed higher adsorption on crushed natural carbonate as compared to the three SAILs studied here. This reflects the advantage of using SAILs as surfactants in surfactant flooding instead of the conventional ones.
2. Amount of surface active agent adsorbed on natural carbonate was in the order of CTAB > Pyrrolidinium > pyridinium > Imidazolium.
3. Adsorption isotherm data for all the SAILs and conventional surfactants studied here were successfully analysed by Langmuir, Freundlich, Redlich-Peterson, and Sips adsorption isotherm models. All of them were best fitted with Sips adsorption isotherm models.
4. In this work, a full factorial design was utilised for designing experiments in order to study effect of multiple factors on the amount of SAIL adsorbed. It had been found that for all the SAILs, amount adsorbed increases upon changing the adsorbent from carbonate to sandstone. This behaviour is attributed to the excessive negative charge on sandstone rock. Also, imidazolium based SAIL exhibited minimal adsorption on carbonate rock (0.0708 mg/m^2^).
5. Also adsorption of SAILs upon the two negatively charged adsorbents decreases as concentration of divalent ions, Ca^++^, increases in the formation brine.
6. The basic mechanism for adsorption is evaluated to be due to electrostatic attraction between cationic charged SAILs and negatively charged carbonate and sandstone samples.
7. It can be concluded that imidazolium based SAILs, exhibit low adsorption on carbonate rock at high salinity, which is not observed for conventional cationic and anionic surfactants. Such low adsorption characteristic of imidazolium based SAILs on negatively charged rock surfaces when integrated with its potential to lower IFT between crude oil and water makes it a deserving candidate for surfactant assisted EOR processes.

## Supplementary information

Supplementary information S1
