# Supplementary material for: Adsorption of Surface Active Ionic Liquids on Different Rock Types under High Salinity Conditions
Source: Sci Rep. 2019 Oct 14;9:14760. doi: 10.1038/s41598-019-51318-2 (PMC6791850; doi:10.1038/s41598-019-51318-2)
Supplement: Supplementary file 1 — Supplementary information S1 [file 41598_2019_51318_MOESM1_ESM.docx]

**Supplemenatry Information S1**

**Adsorption of Surface Active Ionic Liquids on Different Rock Types under High Salinity Conditions**

Shilpa Kulbhushan Nandwani ^1^, Mousumi Chakraborty * ^1^ & Smita Gupta * ^1^

^1^ Department of Chemical Engineering, Sardar Vallabhbhai National Institute of Technology, Surat, Gujarat, India.

* Corresponding author: Smita Gupta- [smitagupta12@rediffmail.com](mailto:smitagupta12@rediffmail.com), Mousumi Chakraborty- [mousumi_chakra@yahoo.com](mailto:mousumi_chakra@yahoo.com)

Regression Equation

Adsorption = 6.0636

(mg/m^2^ ) + 2.4909 Type of SAIL_Pyrrolidinium

+ 1.9483 Type of SAIL_Pyridinium

- 4.4392 Type of SAIL_Imidazolium

+ 0.8807 Type of brine_BRINE I

+ 0.1783 Type of brine_BRINE II

- 1.0590 Type of brine_BRINE III

+ 0.6864 Tempertaure_40

- 0.6864 Tempertaure_60

+ 1.9632 Type of adsorbent_sandstone

- 1.9632 Type of adsorbent_carbonate

- 0.031 Type of SAIL*Type of brine_Pyrrolidinium BRINE I

- 0.075 Type of SAIL*Type of brine_Pyrrolidinium BRINE II

+ 0.105 Type of SAIL*Type of brine_Pyrrolidinium BRINE III

+ 0.274 Type of SAIL*Type of brine_Pyridinium BRINE I

+ 0.082 Type of SAIL*Type of brine_Pyridinium BRINE II

- 0.357 Type of SAIL*Type of brine_Pyridinium BRINE III

- 0.244 Type of SAIL*Type of brine_Imidazolium BRINE I

- 0.008 Type of SAIL*Type of brine_Imidazolium BRINE II

+ 0.252 Type of SAIL*Type of brine_Imidazolium BRINE III

- 0.1450 Type of SAIL*Tempertaure_Pyrrolidinium 40

+ 0.1450 Type of SAIL*Tempertaure_Pyrrolidinium 60

+ 0.1805 Type of SAIL*Tempertaure_Pyridinium 40

- 0.1805 Type of SAIL*Tempertaure_Pyridinium 60

- 0.0354 Type of SAIL*Tempertaure_Imidazolium 40

+ 0.0354 Type of SAIL*Tempertaure_Imidazolium 60

+ 0.5629 Type of SAIL*Type of adsorbent_Pyrrolidinium sandstone

- 0.5629 Type of SAIL*Type of adsorbent_Pyrrolidinium carbonate

+ 0.6344 Type of SAIL*Type of adsorbent_Pyridinium sandstone

- 0.6344 Type of SAIL*Type of adsorbent_Pyridinium carbonate

- 1.1974 Type of SAIL*Type of adsorbent_Imidazolium sandstone

+ 1.1974 Type of SAIL*Type of adsorbent_Imidazolium carbonate

- 0.1433 Type of brine*Tempertaure_BRINE I 40

+ 0.1433 Type of brine*Tempertaure_BRINE I 60

+ 0.1654 Type of brine*Tempertaure_BRINE II 40

- 0.1654 Type of brine*Tempertaure_BRINE II 60

- 0.0220 Type of brine*Tempertaure_BRINE III 40

+ 0.0220 Type of brine*Tempertaure_BRINE III 60

+ 0.2764 Type of brine*Type of adsorbent_BRINE I sandstone

- 0.2764 Type of brine*Type of adsorbent_BRINE I carbonate

+ 0.0521 Type of brine*Type of adsorbent_BRINE II sandstone

- 0.0521 Type of brine*Type of adsorbent_BRINE II carbonate

- 0.3285 Type of brine*Type of adsorbent_BRINE III sandstone

+ 0.3285 Type of brine*Type of adsorbent_BRINE III carbonate

+ 0.0156 Tempertaure*Type of adsorbent_40 sandstone

- 0.0156 Tempertaure*Type of adsorbent_40 carbonate

- 0.0156 Tempertaure*Type of adsorbent_60 sandstone

+ 0.0156 Tempertaure*Type of adsorbent_60 carbonate
